# Calcineurin Broadly Regulates the Initiation of Skeletal Muscle-Specific Gene Expression by Binding Target Promoters and Facilitating the Interaction of the SWI/SNF Chromatin Remodeling Enzyme

**DOI:** 10.1128/MCB.00063-19

**Published:** 2019-09-11

**Authors:** Hanna Witwicka, Jumpei Nogami, Sabriya A. Syed, Kazumitsu Maehara, Teresita Padilla-Benavides, Yasuyuki Ohkawa, Anthony N. Imbalzano

**Affiliations:** a Department of Biochemistry and Molecular Pharmacology, University of Massachusetts Medical School, Worcester, Massachusetts, USA; b Division of Transcriptomics, Medical Institute of Bioregulation, Kyushu University, Higashiku, Fukuoka, Japan

**Keywords:** Brg1, SWI/SNF, calcineurin, chromatin remodeling, differentiation, gene regulation, myogenesis

## Abstract

Calcineurin (Cn) is a calcium-activated serine/threonine protein phosphatase that is broadly implicated in diverse cellular processes, including the regulation of gene expression. During skeletal muscle differentiation, Cn activates the nuclear factor of activated T-cell (NFAT) transcription factor but also promotes differentiation by counteracting the negative influences of protein kinase C beta (PKCβ) via dephosphorylation and activation of Brg1, an enzymatic subunit of the mammalian SWI/SNF ATP-dependent chromatin remodeling enzyme. Here we identified four major temporal patterns of Cn-dependent gene expression in differentiating myoblasts and determined that Cn is broadly required for the activation of the myogenic gene expression program. Mechanistically, Cn promotes gene expression through direct binding to myogenic promoter sequences and facilitating the binding of Brg1, other SWI/SNF subunit proteins, and MyoD, a critical lineage determinant for skeletal muscle differentiation. We conclude that the Cn phosphatase directly impacts the expression of myogenic genes by promoting ATP-dependent chromatin remodeling and formation of transcription-competent promoters.

## Abstract (precis)

Calcineurin (Cn) is a calcium-activated serine/threonine protein phosphatase that is broadly implicated in diverse cellular processes, including the regulation of gene expression.

## INTRODUCTION

Myoblast differentiation is an essential process during skeletal muscle development where mononuclear myoblasts withdraw from the cell cycle and undergo fusion and other morphological changes to form multinucleated myotubes. This process is coordinated by the family of myogenic regulatory factors (MRFs), whose members include MyoD, myogenin, Myf5, and Mrf4, in cooperation with the mouse embryonic fibroblast (MEF) family of transcription factors and other auxiliary transcriptional regulators. MRFs regulate the commitment, determination, and differentiation of skeletal muscle progenitor cells. The ability of MRFs to drive the myogenic gene expression needed for differentiate requires remodeling of chromatin at target genes, which depends on the recruitment of histone modifying and chromatin remodeling complexes that alter nucleosome structure and the local chromatin environment (1–3).

The SWI/SNF (switch/sucrose nonfermentable) complexes are large, multiprotein, ATP-dependent chromatin remodeling enzymes (4–6) that alter nucleosome structure to promote transcription, replication, recombination, and repair (7–10). The chromatin remodeling activity of the mammalian SWI/SNF enzyme is required for the initiation of many developmental and differentiation programs (11–14), including activation of myogenic genes upon differentiation signaling (15, 16). Mammalian SWI/SNF complexes contain one of two related ATPase subunits, i.e., either Brahma related gene 1 (Brg1) or Brahma (Brm), and a collection of at least 9 to 12 associated proteins known as Brg1/Brm-associated factors (Bafs) (4, 17, 18). Mammalian SWI/SNF enzyme function can be influenced by the assembly of different combinations of Baf subunits around the different ATPases (12, 19). Furthermore, signal transduction pathways promote specific posttranslational modifications of SWI/SNF subunit proteins that influence enzyme activity (15, 20–23). In skeletal muscle differentiation, p38 mitogen-activated protein kinase (MAPK) phosphorylates the Baf60c subunit, which then allows the recruitment of the rest of SWI/SNF remodeling complex to myogenic promoters (24). Our group previously showed that casein kinase 2 (CK2) phosphorylates Brg1 to regulate *Pax7* expression and to promote myoblast survival and proliferation (21) and that protein kinase C β1 (PKCβ1) phosphorylates Brg1, which represses chromatin remodeling function and, consequently, myogenesis (20).

Calcineurin (Cn) is a serine/threonine phosphatase that is regulated by changes in the intracellular concentration of Ca^2+^ (25). Cn is a heterodimer formed by an association of catalytic subunit A (CnA) and regulatory subunit B (CnB) (26, 27). Its mechanism of action has been characterized extensively in lymphocytes, where activated Cn dephosphorylates nuclear factor of activated T-cell (NFAT) transcription factors. Dephosphorylated NFAT translocates to the nucleus and binds to promoter regions of target genes to regulate gene expression (28–31). In skeletal muscle, Cn-dependent binding of NFAT to target promoters controls skeletal muscle fiber type and primary muscle fiber number during development (32, 33) and growth (34–38) of multinucleated muscle cells. Cn is also required for the initiation of skeletal muscle differentiation (39), by mechanisms that are independent of NFAT (40, 41). More recently, we reported novel functions for Cn in chromatin remodeling. We showed that Cn is bound to Brg1 at the myogenin promoter and that it dephosphorylates Brg1 shortly after cells start the differentiation process to positively promote differentiation (20).

The aim of the current study was to explore the global effect of Cn on gene expression in myoblasts. We demonstrate that inhibition of Cn in myoblasts globally downregulates expression of genes important for muscle structure and function. We identified four major temporal patterns of Cn-dependent gene expression. Mechanistically, we show that Cn acts as a chromatin binding regulatory protein, interacting with Brg1 to facilitate SWI/SNF enzyme and MyoD binding to myogenic gene regulatory sequences.

## RESULTS

### Transcriptome sequencing (RNA-seq) identification of genes differentially expressed in myoblasts treated with Cn inhibitor FK506.

**FIG 1:**
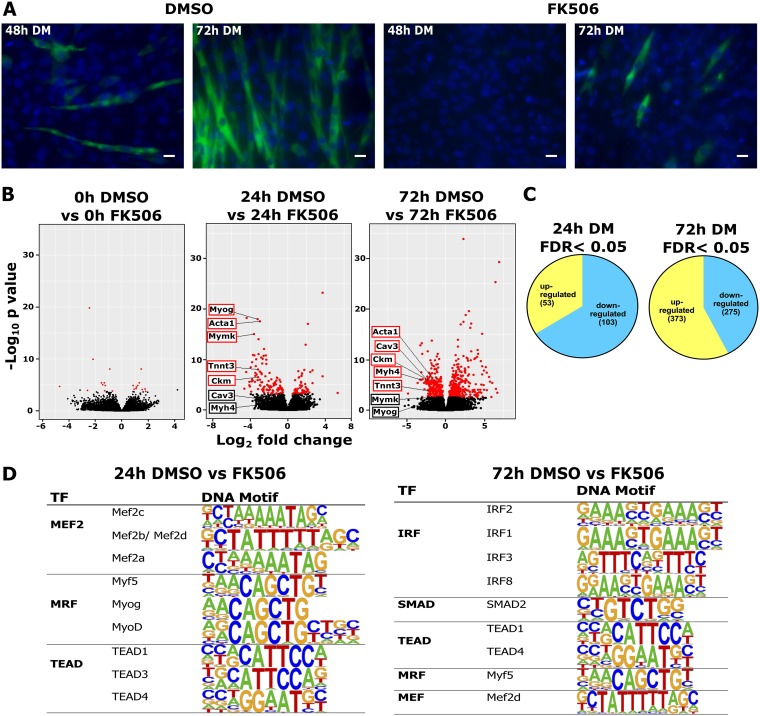
Differential gene expression in myoblasts treated with Cn inhibitor. (A) Myotube formation in differentiating C2C12 cells is inhibited by FK506 treatment. The cells were fixed and analyzed by immunofluorescence staining using the anti-myosin heavy-chain monoclonal Ab MF20 (green). The nuclei were visualized by DAPI staining (blue). Scale bars, 20 μm. (B) Volcano plots displaying differentially expressed genes between control (DMSO)-treated and Cn inhibitor (FK506)-treated differentiated C2C12 cells. The y-axis data correspond to the mean log_10_ expression levels (*P* values). The red dots represent the up- and downregulated transcripts between DMSO-treated and FK506-treated samples (false-discovery rate [FDR] of <0.05). The black dots represent the expression levels of transcripts that did not reach statistical significance (FDR of >0.05). (C) A Venn diagram displaying the number of genes upregulated and downregulated by FK506 treatment at 24 and 72 h postdifferentiation. (D) Transcription factor (TF) binding motifs identified within 1 kb upstream of the TSS of genes differentially expressed in cells differentiated for 24 and 72 h in the presence of FK506. The motifs displayed had *P* values of ≤0.001.

To better understand the involvement of Cn in the gene regulation program underlying myogenesis, we compared the transcriptomes of C2C12 myoblasts at three time points of differentiation (0, 24, and 72 h after induction of differentiation) in cells treated with the Cn inhibitor FK506 to those of control cells. FK506 inhibits the phosphatase activity of Cn by binding to the immunophilin FKB12; the drug-bound FKB12 binds to and blocks Cn function (28). C2C12 myoblasts treated with FK506 showed significant inhibition of myotube formation at the 48-h and 72-h time points compared to the dimethyl sulfoxide (DMSO) control, confirming prior results demonstrating that inhibition of Cn activity blocked myoblast differentiation (39–41) (Fig. 1A). Myogenic gene expression is temporally regulated, with different genes expressed with different kinetics during differentiation (42–44). We analyzed gene expression 24 and 72 h after the induction of differentiation to distinguish the impacts of Cn inhibition on genes expressed at early or late times of myogenesis.

At 24 h postdifferentiation, 156 genes were differentially expressed in the presence of FK506 (false discovery rate [FDR] < 0.05). Fewer genes were upregulated (*n* = 53) than were downregulated (*n* = 103). At the later time point of differentiation (72 h), 648 genes were differentially expressed. More genes were upregulated (373) than were downregulated (275) (Fig. 1B and C). In confluent myoblasts at the onset of differentiation (0 h), only 21 genes were differentially expressed in the presence of FK506. The complete list of differentially expressed genes determined by RNA-seq analysis is shown in Table S1 in the supplemental material. Specific myogenic genes were identified and are labeled on the volcano plots in Fig. 1B. The results indicate that myogenic genes are downregulated by Cn inhibition at the 24-h and 72-h time points. Review of these genes determined that the genes largely encoded structural and functional proteins expressed during differentiation. Specifically, the levels of expression of genes encoding the regulatory proteins MyoD, Myf5, Mef2A, and Mef2D were unaffected by Cn inhibition by FK506, whereas the genes encoding myogenin, Mef2C, and Six1 were seen to be sensitive to the Cn inhibitor. The MRF4 regulator was not expressed. In addition, we noted that none of the genes encoding NFAT proteins and none of the genes encoding mammalian SWI/SNF subunits showed expression changes due to Cn inhibition by FK506 (Table S1).

We performed *de novo* motif analysis on the differentially expressed genes. The most significantly enriched motifs seen at 24 h postdifferentiation were those for Mef2 transcription factors and E-boxes bound by the MRFs Myf5, myogenin, and MyoD, and we noted the continued presence of MRF and Mef2 protein binding sites at Cn-regulated genes at late times of differentiation (Fig. 1D). These findings indicate that genes that require Cn for expression are also regulated by myogenic regulatory factors, strongly reinforcing the idea of a connection between Cn and expression of the myogenic gene program.

**FIG 2:**
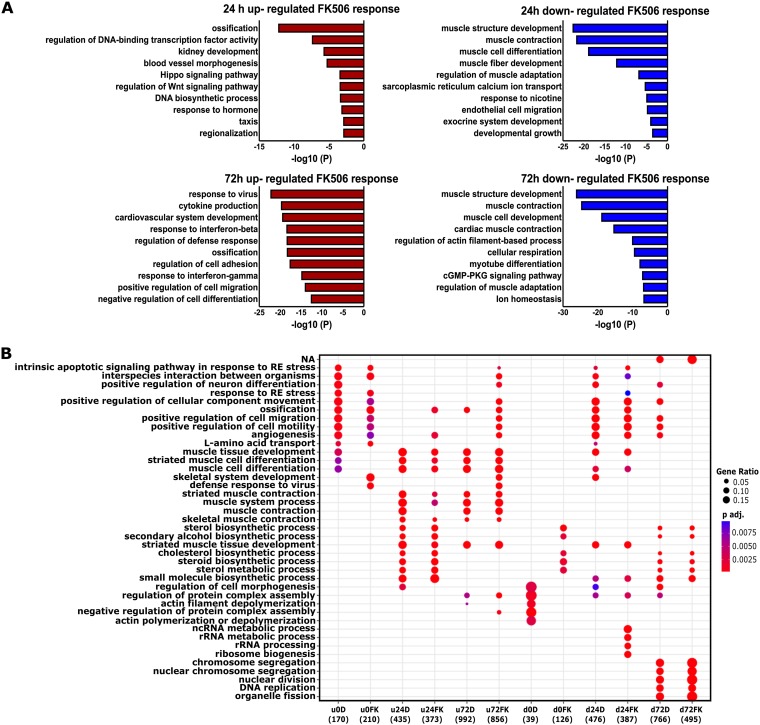
Enriched GO term analysis of differentially expressed genes. (A) Gene ontology analyses on genes differentially expressed by FK506 treatment for 24 and 72 h postdifferentiation. (B) GO analysis of the biological processes shows differences between treatments (DMSO versus FK506) in muscle-related categories. NA, not applicable; ncRNA, noncoding RNA; p adj., adjusted *P* value; RE, endoplasmic reticulum; u, upregulated; d, downregulated; D, DMSO treated; FK, FK506 treated.

The top hits from a gene ontology (GO) analysis of genes upregulated and downregulated by Cn inhibition at the 24-h and 72-h time points are shown in Fig. 2. GO terms identified by side-by-side comparisons of upregulated and downregulated genes at each time point are presented in Fig. 2B. Genes related to muscle differentiation and function were downregulated in cells treated with FK506 (Fig. 2). In contrast, we observed upregulation of genes associated with ossification, responses to interferons and viruses, and cardiovascular and blood vessel development. These results are consistent with documented roles for Cn in the regulation of NFAT function in these processes (45–47). These results are also consistent with our finding that the most significantly enriched DNA binding motif at 72 h postdifferentiation was the interferon regulatory factor (IRF) binding sequence (Fig. 1D) and with findings indicating that IRFs regulate expression of the vascular cell adhesion molecule 1 (VCAM-1) receptor that mediates cell-cell adhesion and is important for myoblast fusion (48, 49).

### Global expression analysis reveals four major temporal gene expression patterns that are dependent on Cn.

**FIG 3:**
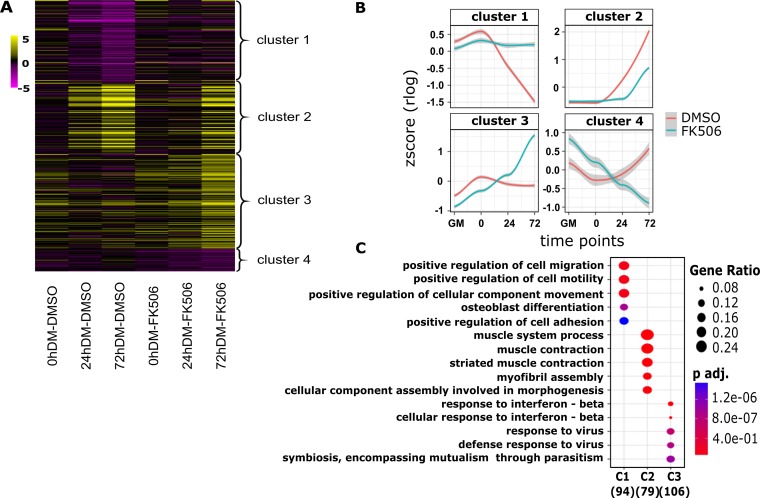
Cluster analysis of differentially expressed genes at three differentiation time points in myoblasts treated with Cn inhibitor. (A) Heat map comparing differential expression levels of 308 FK506 treatment-specific genes, categorized in four different clusters. Each column represents an experimental sample (times 0, 24, and 72 h in differentiation medium [DM]) compared to the proliferating myoblast sample cultured in growth medium (GM). Each row represents a specific gene. The colors range from yellow (high expression) to magenta (low expression) and represent the relative log_2_ ratios of expression levels. (B) Kinetic expression patterns of the four clusters of genes. Lines represent the LOESS (locally estimated scatterplot smoothing) fitting for the relative expression levels of all genes in the cluster. The gray shading represents 95% confidence intervals. (C) Gene ontology analysis of differentially expressed genes within cluster 1 (C1), C2, and C3 identified the top enriched GO terms with the corresponding enrichment *P* values and gene ratios.

**FIG 4:**
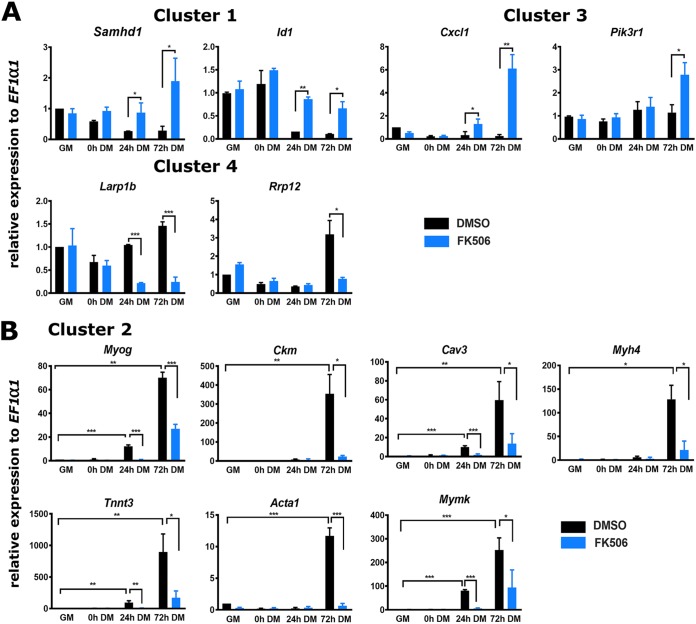
Cn regulates expression of myogenic genes and other genes during myoblast differentiation. (A) Real-time RT-PCR analysis validated expression changes of genes from clusters 1, 3, and 4 in FK506-treated cells. (B) RT-qPCR showed that expression of cluster 2 genes *Myog*, *Ckm*, *Myh4*, *Mymk*, *Tnnt3*, *Acta1*, and *Cav3* was downregulated in FK506-treated C2C12 cells. GM, proliferating cells in growth medium; DM, differentiation medium used for growth for the indicated times. Data represent averages of results from three or more independent samples performed in duplicate and are presented as means ± standard deviations (SD). The level of expression in DMSO-treated GM samples was set to a value of 1, and other values are relative to that sample value. *, *P* ≤ 0.05; **, *P* ≤ 0.001; ***, *P* ≤ 0.0001 (versus GM or vehicle by Student's *t* test).

We continued our analysis by identifying the groups of genes that showed treatment-specific changes in gene expression over time. In total, 308 genes were identified. We analyzed the expression patterns of these differentially expressed genes using clusterProfiler (50). Genes differentially expressed over time were clustered into 4 major groups by k-means clustering, and the data are graphically presented as a heat map (Fig. 3A) and as expression kinetics (Fig. 3B). We performed GO analysis for the differentially expressed genes in these 4 clusters to gain additional insight into the biological processes regulated by Cn during myoblast differentiation (Fig. 3C). Cluster 1 included 95 genes that were downregulated during differentiation but were unchanged in the presence of the Cn inhibitor. GO terms significantly enriched for this group of differentially expressed genes were related to cell migration, motility, and adhesion. In addition, cluster 1 included Inhibitor of differentiation (Id) proteins Id1, Id2, and Id3 (Table S2). Id proteins interact with MyoD and related MRFs prior to differentiation to repress transcription activation activity. The Id1 and Id3 genes have previously been identified as genes repressed by Cn during the activation of skeletal muscle cell differentiation (41). Cluster 2 contained 80 genes whose expression was upregulated during myoblast differentiation but was inhibited or delayed in the presence of FK506. GO terms significantly enriched for cluster 2 genes were related to muscle structure and function. Cluster 3 was composed of 108 genes that were expressed at relatively constant levels across the differentiation time course but that were significantly upregulated in the presence of FK506 expression. GO analysis of cluster 3 genes identified genes implicated in immune response of cells. Cluster 4 had 25 genes with an unusual profile. These genes were upregulated in the presence of FK506 in proliferating cells and in cells at the onset of differentiation but became downregulated as differentiation advanced. No specific GO terms were identified for cluster 4. A complete list of genes in each of the clusters with their log fold change and FDR values is shown in Table S2. These data show that Cn regulates multiple and diverse groups of genes during myoblast differentiation. We measured mRNA expression levels of two randomly chosen genes from each of clusters 1, 3, and 4 by reverse transcription-quantitative PCR (RT-qPCR) analysis (Fig. 4A) to confirm the gene expression kinetics revealed by the cluster analysis.

For further analysis, we focused on genes that were downregulated in the presence of FK506 and are important for muscle structure and/or function (Fig. 1B). We first measured the mRNA expression levels of several such genes to validate RNA-seq and cluster analysis results; *Myog*, *Ckm*, *Myh4*, *Mymk*, *Tnnt3*, *Acta1*, and *Cav3* were analyzed by RT-qPCR. As expected, the expression levels of each of these genes were increased over the differentiation time course and each was significantly downregulated by exposure to Cn inhibitor FK506 (Fig. 4B). These results confirmed that inhibition of Cn during myoblast differentiation impairs expression of multiple myogenic genes whose expression is normally activated during differentiation.

### Inhibition of Cn function prevents its binding to myogenic promoters.

We previously showed that Cn associates with Brg1 and binds to the myogenin promoter early after the start of the differentiation process (20). We hypothesized that the same model might be true for other myogenic genes. Alternatively, Cn may directly regulate the myogenin promoter but indirectly regulate other downstream genes and may mediate the process via the dependency of other genes on myogenin for completing the differentiation process (51, 52).

**FIG 5:**
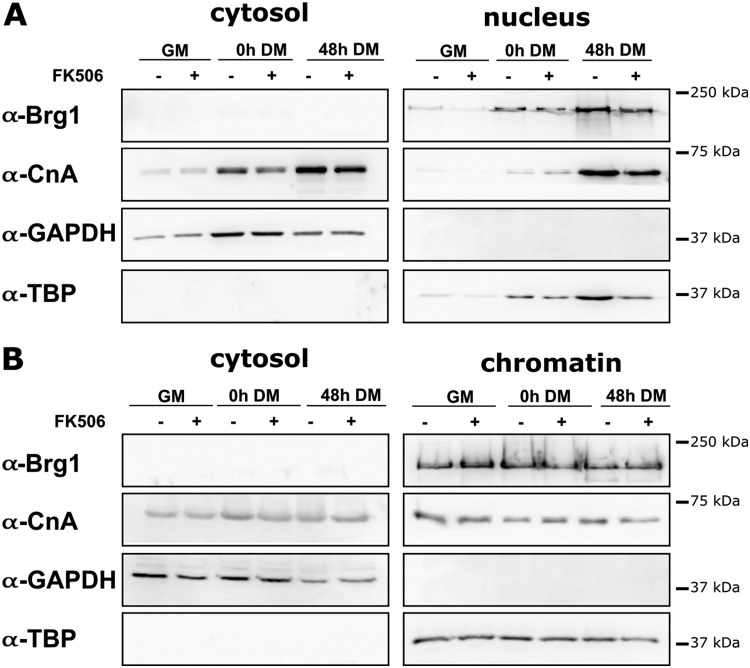
FK506 treatment did not affect intracellular localization (A) or chromatin localization (B) of Cn and Brg1. Representative Western blots of CnA, Brg1, and marker proteins in cytosolic, nuclear, and chromatin fractions derived from proliferating and differentiating myoblasts are shown. GM, proliferating cells in growth medium. DM, differentiation medium used for growth for the indicated times.

We first determined whether the Cn inhibitor could alter the nuclear localization or chromatin association of Cn or Brg1. Immunoblot analyses of subcellular fractions are shown in Fig. 5. Cn was present in both the cytosolic and nuclear fractions as well as in chromatin, and its localization was not changed when cells were treated with FK506. Brg1 was associated only with the nuclear and chromatin fractions and, similarly to Cn, its localization was not affected by FK506. Immunoblots of control proteins show the purity of the fractions. The TATA binding protein (TBP) was found to be associated with the chromatin and the nuclear fractions, while GAPDH (glyceraldehyde-3′-phosphate dehydrogenase) was located only in the cytosolic fraction. These data demonstrate that the effects of inhibition of Cn activity are not due to gross mislocalization of Cn or Brg1 within the cell.

**FIG 6:**
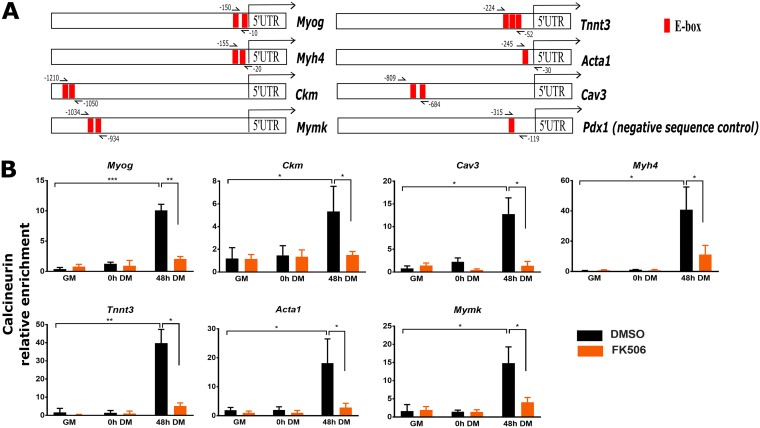
Cn binds to E-box-containing regulatory sequences of myogenic genes during myoblast differentiation. Cn binding is reduced by FK506 treatment. (A) A schematic diagram of regulatory sequences of myogenic genes and localization of E-boxes and primers used in ChIP assays. (B) ChIP assays were performed for Cn binding in C2C12 cells. GM, proliferating cells in growth medium; DM, differentiation medium used for growth for the indicated times. Relative enrichment was defined as the ratio of amplification of the PCR product normalized to control IgG and is shown relative to amplification of a nonspecific control promoter region (*Pdx1*). The data represent averages of results from at least 3 independent experiments performed in triplicate ± SD. *, *P* ≤ 0.05; **, *P* ≤ 0.01; ***, *P* ≤ 0.001 (versus GM or vehicle by Student's *t* test).

We performed chromatin immunoprecipitation (ChIP) assays on the same panel of myogenic genes that were examined in Fig. 4B. PCR primers for ChIP were designed to amplify the E-box-containing regions that bind to the MyoD- and MyoD-related lineage-determining factors (53) within 1.5 kb upstream of the transcriptional start site (TSS) of each gene (Fig. 6A) and that regulate gene expression (53–61). The promoter of the *Pdx1* gene, which encodes the pancreatic and duodenal homeobox 1 transcriptional activator, served as a negative-control sequence for all ChIP experiments, as was done in prior work (62). The *Pdx1* E box is bound by members of the upstream (USF) family of transcription factors (63) and is not bound by MyoD or related factors in proliferating or differentiating myoblasts (62). *Pdx1* is expressed in pancreas, duodenum, gallbladder, stomach, and small intestine (64). Our RNA-seq data confirmed that *Pdx1* was not expressed in proliferating or differentiating myoblasts.

Cn did not interact with regulatory sequences of myogenic genes in proliferating myoblasts (cultured in growth medium [GM]), was weakly bound or not bound in undifferentiated cells at the start of differentiation (i.e., at 0 h in differentiation medium [0 h DM]), and was bound at all myogenic gene promoters in differentiated (48 h DM) cells (Fig. 6B). FK506 treatment of cells greatly reduced Cn binding. We conclude that Cn binding to myogenic promoters is a general occurrence and that FK506-mediated inhibition of Cn function impairs its ability to interact with target gene promoters. The results suggest that Cn is a direct regulator of many myogenic genes.

### Cn inhibition blocks the interaction of the ATPase Brg1 and other subunits of the mammalian SWI/SNF complex with myogenic promoters.

**FIG 7:**
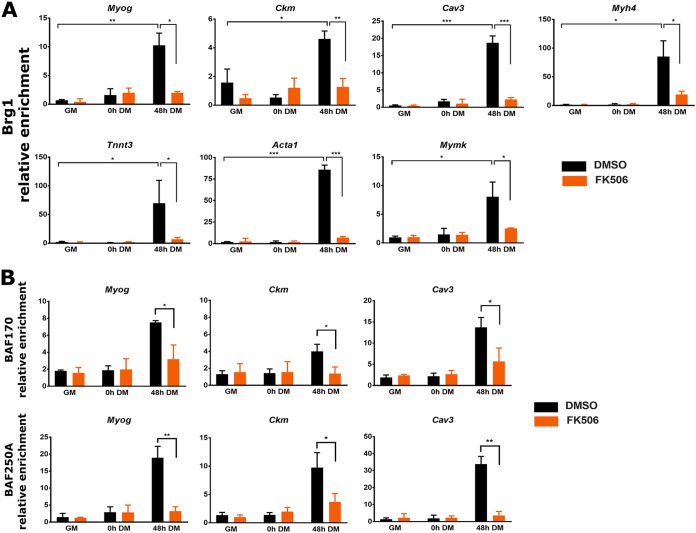
Cn inhibition reduces binding of the SWI/SNF subunit Brg1 (A) and of subunits Baf170 and Baf250A (B) to E-box-containing regulatory sequences of myogenic genes during myoblast differentiation. ChIP assays were performed for Brg1, Baf170, and Baf250A binding in C2C12 cells. GM, proliferating cells in growth medium. DM, differentiation medium used for growth for the indicated times. Relative enrichment was defined as the ratio of amplification of the PCR product normalized to control IgG and is shown relative to amplification of a nonspecific control promoter region (*Pdx1*). The data represent averages of results from at least 3 independent experiments performed in triplicate ± SD. *, *P* ≤ 0.05; **, *P* ≤ 0.01; ***, *P* ≤ 0.001 (versus GM or vehicle by Student's *t* test).

We also performed ChIP assays to determine whether Brg1 recruitment to myogenic regulatory sequences was dependent on Cn. We observed recruitment of Brg1 to regulatory sequences in differentiated (48 h DM) cells at all the tested myogenic genes. In cells treated with FK506, Brg1 binding was significantly diminished at all promoters (Fig. 7A). A subset of the myogenic promoters was tested for binding of Baf170 and of Baf250A/Arid1A, which are other subunits of the mammalian SWI/SNF enzyme complex. The results show that binding of these other subunits paralleled Brg1 binding in that Cn inhibition blocked interactions of these proteins with the promoters (Fig. 7B). These findings indicate that Cn activity is necessary for the interaction of mammalian SWI/SNF chromatin remodeling complexes with regulatory sequences of myogenic genes.

### Mutation of Brg1 sites of Cn activity prevents its interaction with myogenic gene promoters.

**FIG 8:**
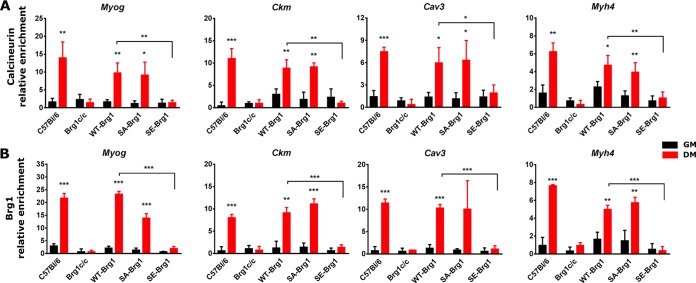
Phosphomimetic mutation of Brg1 amino acids that are dephosphorylated by Cn reduces binding of Cn (A) and Brg1 (B) to the indicated myogenic promoters in differentiating myoblasts. ChIP assays were performed for Cn or Brg1 binding in primary mouse myoblasts (C57BL/6), in primary mouse myoblasts deleted for the gene encoding Brg1 (Brg1c/c), or in primary mouse myoblasts deleted for the gene encoding Brg1 that were expressing wild-type Brg1 (WT-Brg1), Brg1 containing a nonphosphorylatable amino acid at Cn-targeted sites (SA-Brg1), or Brg1 containing a phosphomimetic amino acid at Cn-targeted sites (SE-Brg1). Samples were collected from proliferating cells in growth medium (GM) or at 24 h postdifferentiation (DM). Relative enrichment was defined as the ratio of amplification of the PCR product normalized to control IgG and is shown relative to amplification of a nonspecific control promoter region (*Pdx1*). The data represent averages of results from 3 independent experiments performed in triplicate ± SD. *, *P* ≤ 0.05; **, *P* ≤ 0.01; ***, *P* ≤ 0.001 (by Student’s *t* test).

In previous work, we showed that PKCβ-mediated phosphorylation of Brg1 prior to the onset of differentiation was counteracted by Cn-mediated dephosphorylation and activation of Brg1 immediately after the onset of differentiation (20). Serine amino acids targeted by PKCβ/Cn mapped to the N terminus and C terminus of the Brg1 bromodomain. Mutation of these sites to the phosphomimetic amino acid glutamate (SE) prevented myogenesis, whereas mutation to the nonphosphorylatable amino acid alanine (SA) had no effect on differentiation (20). These experiments used primary myoblasts derived from Brg1-deficient mice that were reconstituted with wild-type Brg1 (WT-Brg1), SA-Brg1, or SE-Brg1. We performed ChIP experiments in differentiating cells and showed that Cn and Brg1 were bound to myogenic promoters in myoblasts expressing WT-Brg1 (Fig. 8). The SE-Brg1 mutant was incapable of binding; the repressive phosphorylation of Brg1 caused by PKCβ was mimicked by the glutamate substitutions, rendering Cn incapable of activating Brg1. As expected, the SA-Brg1 mutant, which cannot be phosphorylated at the relevant PKCβ target amino acids, showed binding of Brg1 and Cn to myogenic regulatory sequences (Fig. 8). These results are consistent with those obtained with the Cn inhibitor, and they reinforce the conclusion that Cn function regulates Brg1 binding to chromatin at myogenic gene regulatory sequences.

### Cn inhibition does not impact the ability of Cn and Brg1 to interact.

**FIG 9:**
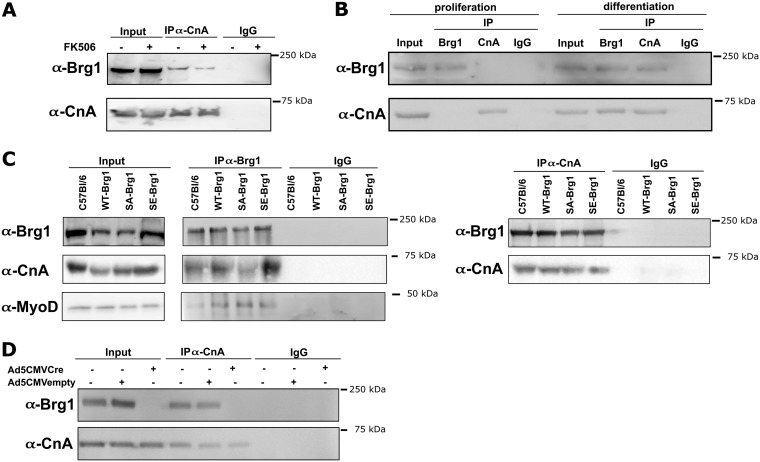
Interactions between Cn and Brg1 are differentiation dependent and are not affected by Cn inhibition or mutation of Brg1 amino acids that are targeted by Cn. (A) Coimmunoprecipitation of Cn and Brg1 from cell lysates from 48 h differentiated C2C12 cells treated with FK506. (B) Coimmunoprecipitation of Cn and Brg1 from cell lysates from proliferating and 24 h differentiated primary mouse myoblasts. (C) Coimmunoprecipitation of Cn and Brg1 and of MyoD and Brg1 from cell lysates from 24 h differentiated primary C57BL/6 myoblasts and from primary mouse myoblasts deleted for the gene encoding Brg1 that were expressing wild-type Brg1 (WT-Brg1), Brg1 containing nonphosphorylatable amino acids at Cn-targeted sites (SA-Brg1), or Brg1 containing phosphomimetic amino acids at Cn-targeted sites (SE-Brg1). (D) Brg1 did not coimmunoprecipitate with Cn from Brg1 conditional myoblasts depleted for Brg1 and subjected to differentiation. Cell lysate from each IP (2.5% of input) served as a loading control. The experiments were performed 3 times, and representative gels are shown.

We previously showed that Cn and Brg1 can be coimmunoprecipitated (co-IP) from cell lysate of differentiating cells (20). Inhibition of Cn function with FK506 had no impact on the ability of these proteins to be isolated in complexes with each other (Fig. 9A). As a complement to this experiment, we looked at the interaction of Cn with Brg1 in proliferating and differentiating myoblasts (Fig. 9B) and in differentiating myoblasts expressing WT-, SA-, or SE-Brg1 (Fig. 9C). Cn interacted with Brg1 only in the differentiating myoblasts. Cn could interact with WT-Brg1 and with the SA-Brg1 and SE-Brg1 mutants (Fig. 9C), despite the observation that SE-Brg1 was not competent for interaction with chromatin. Brg1 can also form a complex with MyoD during differentiation (15, 65). We examined if the Brg1 mutants could interact with MyoD and determined that the Brg1-MyoD interaction was not disrupted by the Brg1 mutations (Fig. 9C). As an additional negative control for our co-IP experiments, we used conditional Brg1 knockout (KO) cells. When Brg1 expression was depleted by Cre recombinase, the Brg1-Cn interaction was not observed, confirming the specificity of the pulldown (Fig. 9D). These results indicate neither inhibition of Cn function nor mutation of the sites of Cn activity on Brg1 affects the interaction that exists between these regulatory proteins. The continued existence of Brg1 protein in the presence of the Cn inhibitor and under conditions in which Cn-targeted residues were mutated to alanine or glutamine suggests that the lack of appropriate phosphorylation or dephosphorylation does not have a significant impact on the steady-state levels of Brg1 and therefore is unlikely to be a major regulator of protein stability.

### Inhibition of Cn blocks MyoD binding to regulatory sequences of myogenic genes.

**FIG 10:**
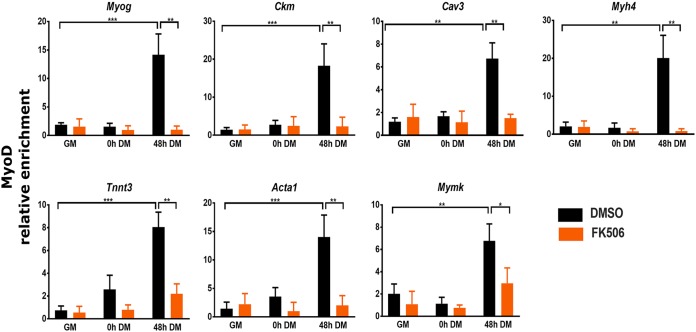
Inhibition of Cn reduced MyoD binding to regulatory sequences of myogenic genes during myoblast differentiation. ChIP assays were performed for MyoD binding in C2C12 cells. GM, proliferating cells in growth medium. DM, differentiation medium used for growth for the indicated times. Relative enrichment was defined as the ratio of amplification of the PCR product normalized to control IgG and is shown relative to amplification of a nonspecific control promoter region (*Pdx1*). The data represent averages of results from at least 3 independent experiments performed in triplicate ± SD. *, *P* ≤ 0.05; **, *P* ≤ 0.01; ***, *P* ≤ 0.001 (versus GM or vehicle by Student's *t* test).

Recruitment of MyoD to myogenic promoters prior to the onset of differentiation can be accomplished by different mechanisms, including gene-specific mechanisms (24, 65, 66). Our RNA-seq data and our prior RT-qPCR analysis (20) showed that MyoD expression was not changed by FK506 treatment. The continued presence of MyoD on myogenic promoters after the onset of differentiation requires the Brg1 ATPase (15, 65). We therefore predicted that the inhibition of Cn would affect the interaction of MyoD with myogenic gene regulatory sequences. We assessed MyoD enrichment at the regulatory sequences of tested genes by ChIP. As shown in Fig. 10, we observed enhanced enrichment of MyoD at all analyzed gene promoters in differentiated cells compared to the level of enrichment seen prior to or at the onset of differentiation. Recruitment of MyoD at these regulatory sequences was attenuated by the Cn inhibitor. These results support the conclusion that Cn is necessary for the stable binding of MyoD to the myogenic gene regulatory sequences during differentiation.

### shRNA-mediated knockdown of Cn impaired myoblast differentiation, affected gene expression, and blocked binding of Cn, Brg1, and MyoD to myogenic regulatory sequences.

**FIG 11:**
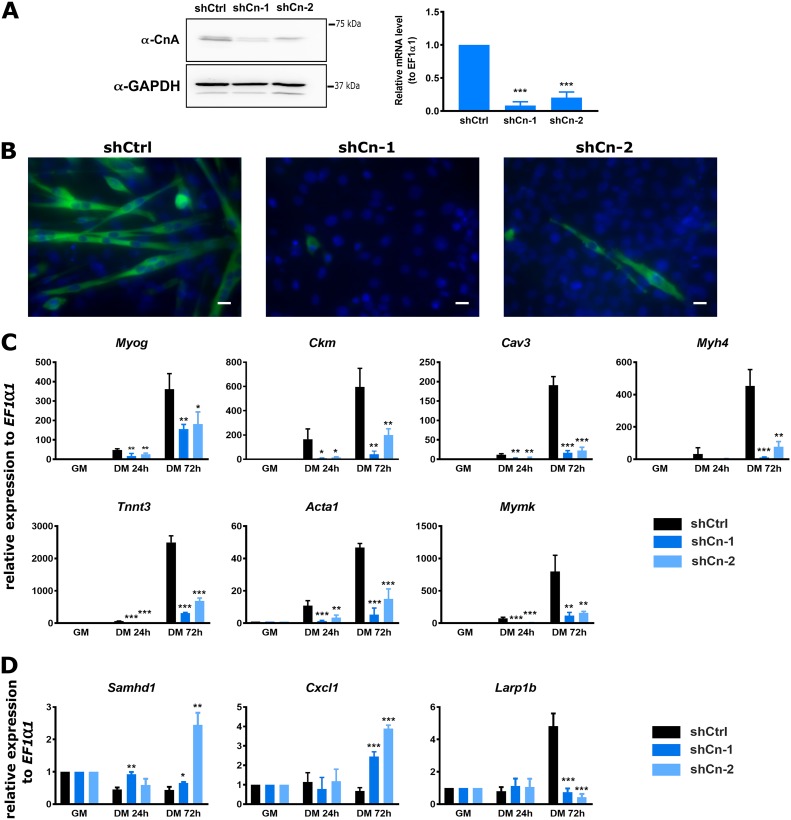
Knockdown of Cn recapitulates findings made with Cn inhibitors and mutation of Brg1 amino acids that are targeted by Cn. (A) Western blot and RT-qPCR analysis showed shRNA-mediated knockdown of endogenous CnA in proliferating C2C12 cells. A scramble shRNA construct (shCtrl) was used as a control. (B) Representative images of myosin heavy-chain staining in 72 h differentiated cells expressing the control or Cn-targeting shRNAs. The cells were fixed and analyzed by immunofluorescence using anti-myosin heavy-chain mAb MF20 (green). The nuclei were visualized by DAPI staining (blue). Scale bars, 20 μm. (C) RT-qPCR showed that expression of *Myog*, *Ckm*, *Myh4*, *Cav3*, *Mymk*, *Tnnt3*, and *Acta1* was downregulated in Cn knockdown cells. GM, proliferating cells in growth medium. DM, differentiation medium used for growth for the indicated times. (D) RT-qPCR analysis validated expression of a representative gene from RNA-seq analysis clusters 1, 3, and 4 and confirmed that the expression levels were changed in Cn knockdown cells compared to the scramble shRNA control. Data represent averages of results from three independent samples in experiments performed in duplicate and are presented as means ± SD. The level of expression in scramble shRNA-expressing cells (A) or GM samples (C and D) was set to a value of 1, and other values are relative to those sample values. *, *P* ≤ 0.05; **, *P* ≤ 0.001; ***, *P* ≤ 0.0001 (by Student's *t* test).

The combination of an inhibitor of Cn activity and mutation of residues on Brg1 that are Cn substrates gave consistent results with regard to the mechanism of Cn function during myogenic gene expression. Nevertheless, to provide additional confirmation, we reduced overall Cn levels by short hairpin RNA (shRNA)-mediated targeting and repeated all of the experiments. Two distinct shRNAs specific for the catalytic subunit of calcineurin A (*Ppp3ca*) were used. shCn-1 targets the 3′ untranslated region (3′UTR), and shCn-2 targets the coding sequence. Cells infected with lentivirus expressing either of the shRNAs targeting Cn or the control shRNA (shCtrl) were selected by the use of puromycin and then evaluated for Cn expression and differentiation of cells (Fig. 11A). The shCtrl had no effect on Cn expression and differentiation. The results seen with both shCn-1 and shCn-2 showed significant reduction of Cn protein and mRNA expression levels compared to the control. Myoblast differentiation was evaluated by myosin heavy chain staining (Fig. 11B); we observed significant reductions in myotube formation in 72-h differentiated cells. Consistent with the reduction of Cn protein levels and lack of differentiation, the levels of mRNA expression of myogenic genes *Myog*, *Ckm*, *Cav3*, *Myh4*, *Tnnt3*, *Acta1*, and *Mymk* (cluster 2 genes from RNA-seq analysis) were significantly reduced in the Cn knockdown cells (Fig. 11C). Representative genes from clusters 1, 3, and 4 (*Smahd1* [cluster 1], *Cxcl1* [cluster 3], and *Larp1b* [cluster 4]) were also affected by knocking down Cn (Fig. 11D).

**FIG 12:**
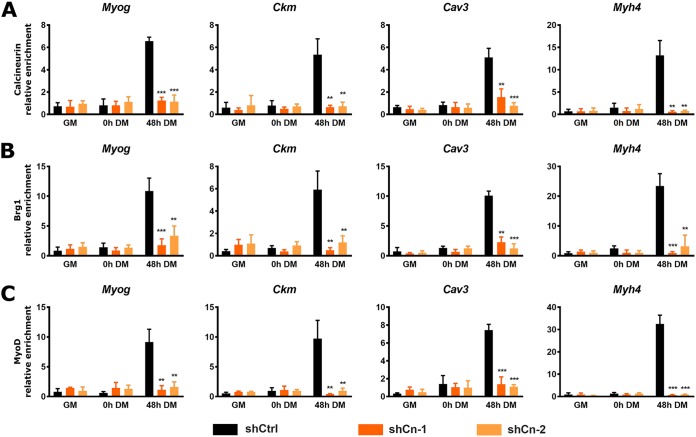
Cn knockdown blocked binding of Cn (A), Brg1 (B), and MyoD (C) to E-box-containing regulatory sequences of myogenic genes during myoblast differentiation. ChIP assays were performed for Cn, Brg1, and MyoD binding in control and Cn knockdown C2C12 cells. Cn was knocked down by the use of two independent shRNA constructs. GM, proliferating cells in growth medium. DM, differentiation medium used for growth for the indicated times. Relative enrichment was defined as the ratio of amplification of the PCR product normalized to control IgG and is shown relative to amplification of a nonspecific control promoter region (*Pdx1*). The data represent averages of results from at least 3 independent experiments performed in triplicate ± SD. *, *P* ≤ 0.05; **, *P* ≤ 0.01; ***, *P* ≤ 0.001 (by Student’s *t* test).

Next, we analyzed recruitment of Cn, Brg1, and MyoD to four of the previously interrogated myogenic regulatory sequences (Fig. 12). As expected, all three proteins were bound to the indicated regulatory sequences in the shCtrl differentiated cells. Cn binding to the regulatory sequences in the Cn knockdown cells was impaired (Fig. 12A). Brg1 binding and MyoD binding (Fig. 12B and C) were also impaired in the Cn knockdown cells, which demonstrates that the reduction in Cn blocked the interaction of these protein with the myogenic regulatory sequences. These results further support the conclusion that Cn promotes myoblast differentiation by binding to myogenic regulatory sequences, facilitating binding of the Brg1 chromatin remodeling enzyme and the stable binding of MyoD and contributing to the activation of the myogenic gene expression program.

## DISCUSSION

### Cn broadly contributes to the activation of the myogenic gene expression program during differentiation.

The data presented here demonstrate that Cn plays a general role in regulating myogenic gene expression during myoblast differentiation. Its mechanism of action is via binding and dephosphorylation of the Brg1 ATPase of the mammalian SWI/SNF chromatin remodeling enzyme, which regulates the ability of Brg1 and other SWI/SNF enzyme subunits to stably associate with myogenic promoters during differentiation. We and others have previously demonstrated that Brg1-based SWI/SNF enzymes remodel chromatin at myogenic regulatory sequences in differentiating cells (15, 16, 65). Failure of Cn to promote association of Brg1 and associated mSWI/SNF enzyme subunits prevents the required enzymatic remodeling of promoter chromatin structure and subsequent gene activation during differentiation.

The consistent observation of Cn binding to each of the myogenic promoters assayed suggests that Cn is directly required for the activation of each target gene. The alternative hypothesis was that Cn was required indirectly via a requirement for activation of myogenin, which is required for activation of myogenic gene products that promote terminal differentiation (67, 68). In prior work examining other cofactors of myogenic gene expression, we determined that the SWI/SNF chromatin remodeling enzyme was required both for the expression of myogenin and for subsequent gene expression (51). These new results spatially link Cn and SWI/SNF enzyme binding to myogenic promoters, which is consistent with Cn function being required for SWI/SNF enzyme function. In contrast, the Prmt5 arginine methyltransferase is required for myogenin activation, but ectopic expression of myogenin promoted myogenic gene expression and differentiation even in the absence of Prmt5 (69), indicating that the requirement for Prmt5 in later stages of the myogenic gene expression cascade was indirect. However, direct evidence that Cn-mediated dephosphorylation of Brg1 must occur while it is bound to promoters is lacking. Future examination of Cn and PKCβ1 binding and function at myogenic regulatory sequences will enable elucidation of mechanistic details concerning the timing, dynamics, and spatial requirements for Brg1 phosphorylation and dephosphorylation.

The promoter binding capability of Cn is a novel function that is poorly understood. There is no evidence that Cn contains a recognized DNA binding domain, raising the possibility that it binds chromatin indirectly. The absence of Cn binding in the presence of the Cn inhibitor suggests the possibility of autodephosphorylation as a necessary prerequisite. However, prior studies indicate that autodephosphorylation is slow and that phosphorylated Cn is more efficiently dephosphorylated by protein phosphatase IIa (70). An alternative hypothesis is that Cn inhibition alters the structure or function of a Cn binding partner, which directly or indirectly results in loss of association with chromatin.

### Regulation of SWI/SNF enzyme by subunit composition and by phosphorylation state.

The diversity of the forms of mammalian SWI/SNF enzyme complex formation is due in part to the existence of several subunits that exclusively or predominantly associate with subsets of enzyme complexes (12, 19). The Baf250A subunit exists in a subset of SWI/SNF complexes known as a SWI/SNF-A or BAF, which contains several unique subunits not found in the other major subfamily of SWI/SNF complexes, referred to as SWI/SNF-B or PBAF complexes (71). The presence of Baf250A at each of the promoters seen in the assays suggests that the A/BAF complex may be the functionally relevant enzyme for promotion of skeletal muscle differentiation.

The literature concerning which of the specific enzyme complexes act(s) at muscle-specific promoters is limited. Brg1 has been identified at many myogenic promoters. Interference with Brg1 function through expression of a dominant-negative enzyme, injection of specific antibodies (Abs), or knockdown blocks myogenic gene expression and differentiation (15, 16, 24, 65, 72), but these data do not distinguish between different types of SWI/SNF enzymes. The Brm ATPase binds to the myogenin promoter in isolated mouse myofibers but not in isolated satellite cells (73). However, knockdown of Brm in cultured myoblasts had a limited effect on differentiation-specific gene expression and instead affected cell cycle withdrawal (72). Those data indicate that A/BAF complex function is a necessary prerequisite for myoblast differentiation. B/PBAF-specific subunits BAF200 and BAF180 are required in mouse heart development (74–76), but Baf250A knockout in mouse neural crest leads to embryonic death due to defective cardiac development (77). Despite the intriguing ramifications of the existence of thousands of different potential SWI/SNF enzyme compositions, comparisons of complexes formed by A/BAF-specific and B/PBAF-specific subunits in the same cell type showed that genomic binding sites and transcriptionally responsive genes largely overlapped, leading to the conclusion that the regulation of gene expression by SWI/SNF enzymes is due to the combined effects of multiple SWI/SNF enzymes (78). Muscle development and differentiation may similarly rely on multiple SWI/SNF enzyme assemblies and may not be attributable to one specific enzyme complex.

Regulation of SWI/SNF chromatin remodeling enzyme activity, via control of the phosphorylation state of different proteins within the enzyme complex, represents an emerging complexity that adds to the alarming complexity posed by the thousands of potential combinatorial assemblies of the enzyme complex from its component subunit proteins (12, 19). Nevertheless, the evidence for this additional layer of regulation is clear. Amino acids N and C terminal to the Brg1 bromodomain are phosphorylated by PKCβ1 in proliferating myoblasts and dephosphorylated by Cn after the onset of differentiation signaling. Failure to remove the phosphorylation prevents remodeling enzyme function and differentiation, while mutation of these amino acids to prevent phosphorylation permits function even in the presence of a Cn inhibitor (20). Here we demonstrated the generality of the requirement for Cn-mediated facilitation of Brg1 function. The PKCβ1/Cn axis is joined by the p38 kinase-mediated phosphorylation of the Baf60c subunit that permits assembly of the SWI/SNF enzyme complex on myogenic promoters (24). Most of the SWI/SNF subunits have been characterized as phosphoproteins (79), suggesting that regulation of activity in response to differentiation signaling may be influenced by other kinases and phosphatases as well.

The consequences of phosphorylation and dephosphorylation of the PKCβ1/Cn-targeted amino acids on Brg1 structure remain to be determined. The fact that Brg1 protein remains present when the relevant amino acids are mutated or when exposed to Cn inhibitor indicates that the phosphorylation state of these amino acids does not render the Brg1 protein unstable. The failure of other SWI/SNF subunits to remain associated with myogenic gene chromatin suggests that the phosphorylation state may control Brg1 protein conformation, interactions with chromatin, interactions with other SWI/SNF enzyme subunits, and/or interactions with other cofactors that contribute to enzyme complex stability or chromatin binding. Additional characterization will improve our understanding of Brg1 and SWI/SNF chromatin remodeling enzyme function in differentiation and may also inform studies on the role of Brg1 in oncogenesis, where Brg1 can be either mutated or overexpressed without mutation in different types of cancer (80, 81).

## MATERIALS AND METHODS

### Antibodies.

Rabbit antisera to Brg1 and MyoD were previously described (65, 82). Pan-calcineurin A (catalog no. 2614), Baf170 (catalog no. 12769), Baf250A (catalog no. 12354), and TBP (catalog no. 8515) antibodies were from Cell Signaling Technologies (Danvers, MA). GAPDH antibody (catalog no. G9295) was from Sigma-Aldrich (St. Louis, MO). MF20 antibody was from the Developmental Studies Hybridoma Bank (Iowa City, IA). Brg1 antibody (G-7; catalog no. sc-17796) was from Santa Cruz Biotechnologies (Santa Cruz, CA) and was used for Western blotting and immunoprecipitation experiments.

### Cell culture.

C2C12 and HEK293T cells were purchased from ATCC (Manassas, VA) and were maintained at subconfluent densities in Dulbecco’s modified Eagle’s medium (DMEM) supplemented with 10% fetal bovine serum (FBS) and 1% penicillin-streptomycin in a humidified incubator at 37°C in 5% CO_2_. For differentiation, C2C12 cells at >80% confluence were switched to DMEM supplemented with 2% horse serum and 2 μg/ml of bovine insulin (Sigma-Aldrich). FK506 (Cayman Chemical, Ann Arbor, MI) was added to the culture 24 h before initiation of differentiation, and FK506 was maintained in the differentiation medium at 2 μM. Medium containing FK506 was changed every day.

Mouse satellite cells were isolated from leg muscles of 3-to-6-week-old Brg1 conditional mice by the use of Percoll sedimentation followed by differential plating as described previously (20). Mice were housed in the animal care facility at the University of Massachusetts Medical School and used in accordance with a protocol approved by the Institutional Animal Care and Use Committee. Brg1-depleted primary myoblasts expressing wild-type Brg1 (WT-Brg1) or Brg1 mutated at sites of PKCβ1/Cn activity were generated as described previously (20). Primary myoblasts were grown and differentiated as described previously (20) on plates coated overnight in 0.02% collagen (Advanced BioMatrix, San Diego, CA).

### shRNA gene knockdown and lentivirus gene transfer.

Calcineurin A-targeting shRNA in pLKO.1 vector was obtained from Sigma-Aldrich (SHCLNG-NM_008913). The shRNA sequences were as follows: for shRNA 1, CCGGGCCAGGAATTGGATTCAGTTTCTCGAGAAACTGAATCCAATTCCTGGCTTTTTG (TRCN0000081058); for shRNA2, CCGGCGCCAACCTTAACTCCATCAACTCGAGTTGATGGAGTTAAGGTTGGCGTTTTTG (TRCN0000081060). The scrambled shRNA control sequence (CCTAAGGTTAAGTCGCCCTCGCTCGAGCGAGGGCGACTTAACCTTAGG) was generated by Sarbassov et al. (83) and was obtained from Addgene, Cambridge, MA (plasmid catalog no. 1864). The shRNA lentiviral constructs were cotransfected with pLP1, pLP2, and pVSVG packaging vectors (Invitrogen, Carlsbad, CA) into HEK293T cells with Lipofectamine 2000 reagent (Invitrogen). The viral supernatant was harvested after 48 h of incubation and filtered through a 0.45-μm-pore-size syringe filter. To infect C2C12 cells, 2.7 × 10^5^ cells were incubated with 5 ml of the filtered viral supernatant supplemented with 8 μg ml^−1^ of Polybrene (Sigma-Aldrich) for 24 h. The transduced cells were subsequently selected in medium containing 2 μg/ml puromycin (Invitrogen) for 4 days.

### RNA-Seq and data analysis.

Total RNA from C2C12 cells treated with FK506 or with DMSO was isolated from proliferating and differentiating cultures (times zero, 24 h, and 72 h) with TRIzol, and libraries were constructed as described previously (84). Five replicates were performed for each sample except for the 72-h sample treated with DMSO, for which only four replicates were performed. The libraries were sequenced using an Illumina HiSeq 1500 system, and the resulting reads were mapped onto the reference mouse genome (GRCm38) by the use of HISAT2 software (ver. 2.2.6) (85). Counting of reads per gene was performed with HTseq software (ver. 0.6.1) (86) such that duplicates in unique molecular identifiers (UMIs) were discarded. After converting unique UMI counts to transcript counts as described previously (87), differentially expressed genes (those with an adjusted *P* value of <0.1) were extracted by the use of R library DESeq2 (version 1.10.1) (88). The differentially expressed genes and cluster analyses are listed in Table S1 and Table S2 in the supplemental material. Gene ontology (GO) term identification was performed using metascape (http://metascape.org). Cluster gene analysis was performed using clusterProfiler (ver. 3/10.0), a multipurpose, open-source tool designed to identify and compare functional relationships between genes (50). RNA-seq data were deposited at the Gene Expression Omnibus (GEO) database (see below). Motif analysis was performed using HOMER motif discovery software as described previously (89). The promoters of the genes that were differentially expressed upon FK506 treatment were analyzed for the enrichment of known motifs. For each gene, we looked for motif enrichment at locations up to 1 kb upstream of the TSS.

### RT-qPCR gene expression analysis.

RNA was extracted using TRIzol reagent (Invitrogen) and the yield determined by measuring optical density at 260 nm (OD_260_). A 1-μg volume of total RNA was subjected to reverse transcription with a QuantiTect reverse transcription kit (Qiagen, Germantown, MD). The resulting cDNA and Fast SYBR green master mix (Applied Biosystems, Foster City, CA) were used for quantitative PCR. Amplification reactions were performed in duplicate in a 10-μl final volume that included the following: 25 ng of template, 0.3 μM concentrations of primers, and 2× SYBR green master mix. The reaction mixtures were processed in QuantStudio3 (Applied Biosystems) using following primers: *Acta1* forward (5′-CTTCCTTTATCGGTATGGAGTCTGCGG-3′) and reverse (5′-GGGGGGCGATGATCTTCATG-3′); *Cav3* forward (5′-TCAATGAGGACATTGTGAAGGTAGA-3′) and reverse (5′-CAGTGTAGACAACAGGCGGT-3′); *Ckm* forward (5′-CTGTCCGTGGAAGCTCTCAACAGC-3′) and reverse (5′-TTTTGTTGTCGTTGTGCCAGATGCC-3′); *Cxcl1* forward (5′-ACCGAGTCATAGCCACACTC-3′) and reverse (5′-CTCCGTTACTTGGGGACACC-3′); *Eef1A1* forward (5′-GGCTTCACTGCTCAGGTGATTATC-3′) and reverse (5′-ACACATGGGCTTGCCAGGGAC-3′); *Id1* forward (5′-AGGTGAGGCGGCATGTGTTCCAG-3′) and reverse (5′-ACCCTCTACCCACTGGACCG-3′); *Larp1b* forward (5′-CAGTCAAGCAGCCAGAGGAA-3) and reverse (5′-CTCTCCCGTCTCCAACTTCG-3′); *Myh4* forward (5′-GGCTTTGAGATCTTTGACTTCAACACC-3′) and reverse (5′-GAGAAGATGCCCATCGGCTTCTCG-3′); *Mymk* forward (5′-GAGAAGATGCCCATCGGCTTCTCG-3′) and reverse (5′-GTCGGCCAGTGCCATCAGGGA-3′); *Myog* forward (5′-GTCCCAACCCAGGAGATCATTTGCTC-3′) and reverse (5′-CCCACTTAAAAGCCCCCTGCTAC-3′); *Pix3r1* forward (5′-TGGATTTCCTGGGAAGTACGG-3′) and reverse (5′-AAGCCGAAGATCCGTAAGGC-3′); *Ppp3ca* forward (5′-GGTCGGGGTGTGCAGTC-3′) and reverse (5′-ATGGAACGGCTTTCACCACC-3′); *Rrp12* forward (5′-AAGCCGAAGATCCGTAAGGC-3′) and reverse (5′-AGGGGCCTTTTCACCAAACA-3′); Samhd1 forward (5′-GAGCAGCTCATTCGGGTGTA-3′) and reverse (5′-TGTCACCATCCTGTGGCT-3′); and *Tnnt3* forward (5′-TGACAAGCTGAGGGACAAGG-3′) and reverse (5′-TGCTTCTGGGCTTGGTCAAT-3′). The delta threshold cycle value (Δ*C_T_*) was calculated for each gene and represents the difference between the *C_T_* value for the gene of interest and that of the *Eef1A1* reference gene. Fold change values were calculated using the 2*^−^*^ΔΔ^*^CT^* method (90).

### Chromatin immunoprecipitation (ChIP) assay.

Chromatin immunoprecipitation assays were performed as previously described (20, 91), with some modifications. Briefly, cells (4 × 10^6^) were cross-linked with 1% formaldehyde (Ted Pella Inc., Redding, CA) for 10 min at room temperature. After quenching of the formaldehyde with 125 mM glycine for 5 min, fixed cells were washed twice with phosphate-buffered saline (PBS) supplemented with protease inhibitor cocktail and lysed with 1 ml buffer A (10 mM Tris HCl [pH 7.5], 10 mM NaCl, 0.5% NP-40, 0.5 μM dithiothreitol [DTT], and protease inhibitors) by incubation on ice for 10 min. The nuclei were pelleted, washed with 1 ml of buffer B (20 mM Tris HCl [pH 8.1], 15 mM NaCl, 60 mM KCl, 1 mM CaCl_2_, 0.5 μM DTT), and incubated for 30 min at 37°C in the presence of 1,000 gel units of micrococcal nuclease (catalog no. M0247S; NEB, Ipswich, MA) in a 300-μl volume of buffer B. The reaction was stopped by adding 15 μl of 0.5 M EDTA. Nuclei were pelleted and resuspended in 300 μl of ChIP buffer (100 mM Tris HCl [pH 8.1], 20 mM EDTA, 200 mM NaCl, 0.2% sodium deoxycholate, 2% Triton X-100, and protease inhibitors), sonicated for 10 min (medium intensity, 30 s on/30 s off) in a Bioruptor UCD-200 system (Diagenode, Denville, NJ), and centrifuged at 21,000 × *g* for 5 min. The length of the fragmented chromatin was between 200 and 500 bp as analyzed on agarose gels. Chromatin concentrations were measured using a Qubit 3 fluorometer (Invitrogen). After preclearing was performed with protein A-agarose, chromatin (2 to 4 μg) was subjected to immunoprecipitation with the specific antibodies listed above or with anti-IgG as a negative control at 4°C overnight, and immunocomplexes were recovered by incubation with protein A-agarose magnetic beads (Invitrogen). Sequential washes of 5 min each were performed with buffers A to D (buffer A, 50 mM Tris [pH 8.1], 10 mM EDTA, 100 mM NaCl, 1% Triton X-100, and 0.1% sodium deoxycholate; buffer B, 50 mM Tris [pH 8.1], 2 mM EDTA, 500 NaCl, 1% Triton X-100, and 0.1% sodium deoxycholate; buffer C, 10 mM Tris [pH 8.1], 1 mM EDTA, 0.25 M LiCl_2_, 1% NP-40, and 1% sodium deoxycholate; buffer D, 10 mM Tris [pH 8.1] and 1 mM EDTA), and immune complexes were eluted in 100 μl of elution buffer (0.1 M NaHCO_3_, 1% SDS) for 30 min at 65°C, incubated with 1 μl of RNnase A (0.5 mg/ml) for 30 min at 37°C, and reverse cross-linked by addition of 6 μl of 5 M NaCl and 1 μl of proteinase K (1 mg/ml) overnight at 65°C. DNA was purified using a ChIP DNA Clean & Concentrator kit (Zymo Research, Irvine, CA). Bound DNA fragments were analyzed by quantitative PCR using SYBR green master mix. Quantification was performed using the fold enrichment threshold cycle method [2^–(^*^CT^* ^sample –^ *^CT^* ^IgG)^], and data are shown relative to the results determined for a control region, the promoter for the *Pdx1* gene. The primer sequences used were as follows: *Acta1* forward (5′-TGTTGCTGCCCTTCCCAAGCCATATTT-3′) and reverse (5′-GCAGACAGCTGGGGATACTCTCCATAT-3′); *Cav3* forward (5′-CCTAGGTGTCTCAGTCCAGTTA-3′) and reverse (5′-CTGCCACGTAGATCTTGGAAAT-3′); *Ckm* forward (5′-GACACCCGAGATGCCTGGTT-3′) and reverse (5′-GATCCACCAGGGACAGGGTT-3′); *Myh4* forward (5′-CACCCAAGCCGGGAGAAACAGCC-3′) and reverse (5′-GAGGAAGGACAGGACAGAGGCACC-3′); *Mymk* forward (5′-CTGACAGCAGGGTTAGGGCT-3′) and reverse (5′-TGATGTGTACCCTTTCTCCCC-3′); *Myog* forward (5′-ACACCAACTGCTGGGTGCCA-3′) and reverse (5′-GAATCACATGTAATCCACTGG-3′); and *Tnnt3* forward (5′-GCAGCTGACACCTTTCTGGAAC-3′) and reverse (5′-ATTGGCCAGCAGATGGGTGG-3′. The silent gene promoter *Pdx1* was used as a negative control, as described previously (62), with the following primer sequences: forward (5′-GAAGTCCTCCGGACATCTCCCCATACGAAG-3′) and reverse (5′-GGATTTCATCCACGGGAAAGGGAGCTGGAC-3′). The primer positions and locations of E-boxes for all of the regulatory sequences (53–61) are shown schematically in Fig. 6A.

### Immunoprecipitation.

Dishes (100 mm in diameter) of 24-h differentiated C2C12 cells treated with FK506 or with DMSO or 100-mm-diameter dishes of primary myoblasts expressing WT-Brg1 or Brg1 mutants were washed with ice-cold PBS twice and lysed in 0.5 ml of lysis buffer (50 mM Tris-HCl [pH 7.5], 150 mM NaCl, 1% Nonidet P-40, 0.5% sodium deoxycholate, and 1 mM CaCl_2_, supplemented with complete protease inhibitors). Lysates were cleared by centrifugation and precleared with PureProteome protein A magnetic beads (Millipore) for 2 h at 4°C. Next, cell extract was incubated with 10 μl of pan-calcineurin A antibody (Cell Signaling Technologies) or 10 μl of Brg1 antisera overnight at 4°C, followed by incubation with 100 μl with PureProteome protein A mix magnetic beads. After extensive washing of beads with washing buffer (24 mM Tris-HCl [pH 7.5], 300 mM NaCl, 0.5% NP-40, 1 mM CaCl_2_), the precipitated proteins were eluted in Laemmli buffer and detected by Western blot analysis using chemiluminescent detection.

### Immunofluorescence.

Cells grown on coverslips were fixed with 3% paraformaldehyde, permeabilized with 0.5% Triton X-100, and blocked with 1% bovine serum albumin (BSA) solution–PBS. Primary and secondary antibodies were diluted in 1% blocking solution. The primary antibody against myosin heavy chain, monoclonal antibody (MAb) MF20, was used at a dilution of 1:100. Goat anti-mouse secondary antibody conjugated with Alexa Fluor 488 (Jackson Laboratory, West Grove, PA) was applied for 1 h at room temperature. DAPI (4′,6-diamidino-2-phenylindole) staining was used to visualize nuclei. The samples were mounted with ProLong Gold antifade reagent (Life Technologies) and observed with inverted fluorescence microscopy (Leica DMI6000). Leica LAS AF Lite software was used for recording and image processing.

### Subcellular fractionation.

Cell fractionation was performed as described previously (92). Briefly, cell pellets were resuspended in hypotonic buffer A (10 mM HEPES [pH 7.9], 1.5 mM MgCl_2_, 10 mM KCl, 0.5 mM DTT, and protease inhibitor cocktail). The cell suspension was centrifuged, and the supernatant was discarded. Intact pelleted cells were resuspended in buffer A and pushed through a 26-gauge needle to disrupt the cell membrane. The disrupted cell suspension was centrifuged at 10,000 × *g* for 20 min, and the supernatant was saved as the cytosolic fraction. Pelleted nuclei were resuspended in buffer C (20 mM HEPES [pH 7.9], 1.5 mM MgCl_2_, 0.42 M NaCl, 0.2 mM EDTA, 25% [vol/vol] glycerol, 0.5 mM DTT, and complete protease inhibitor cocktail) and disrupted by being pushed 10 times through a 26-gauge needle. The nuclear suspension was centrifuged at 20,000 × *g* for 5 min, and the supernatant was saved as the nuclear extract. Chromatin isolation was performed as described previously (21). Cells were resuspended in cytoskeleton (CSK) buffer {10 mM PIPES [piperazine-*N*,*N*′-bis(2-ethanesulfonic acid); pH 6.8], 100 mM NaCl, 300 mM sucrose, 3 mM MgCl_2_, 1 mM EGTA, 1 mM DTT, 0.5% (vol/vol) Triton X-100, and complete protease inhibitor cocktail}. The cytosolic fraction was separated from soluble proteins by centrifugation, and the pellet was resuspended in CSK buffer. Chromatin was solubilized by DNase I digestion (New England Biolabs) in CSK buffer for 15 min at 37°C. (NH_4_)_2_SO_4_ (0.25 M) was added, the samples were incubated for 5 min at 4°C and centrifuged at 5,000 × *g* for 3 min, and the supernatant was saved as a soluble chromatin fraction.

### Statistical analysis.

All quantitative ChIP and RT-qPCR data are shown as means ± standard deviations (SD) of results from at least three (*n* = 3) biological replicates for each experiment. Statistical analysis was performed using Student’s *t* test in Prism (GraphPad Software Inc.). For all analyses, a *P* value of less than 0.05 was considered to be statistically significant.

### Data availability.

RNA-seq data were deposited at the Gene Expression Omnibus (GEO) database under accession number GSE125914.

## Supplementary Material

Supplemental file 1

Supplemental file 2
